# ELDER MISTREATMENT AND DEPRESSIVE SYMPTOMS AMONG OLDER CHINESE AMERICANS

**DOI:** 10.1093/geroni/igz038.2797

**Published:** 2019-11-08

**Authors:** Ying-Yu Chao, Yu-Ping Chang, XinQi Dong

**Affiliations:** 1 Rutgers School of Nursing, Newark, United States; 2 The State University of New York, University at Buffalo, Buffalo, New York, United States; 3 Rutgers University, New Brunswick, New Jersey, United States

## Abstract

This study aimed to examine the association between different types of elder mistreatment and depressive symptoms among U.S. Chinese older adults. Data were from the Population Study of Chinese Elderly in Chicago (PINE). Participants were 3,157 Chinese older adults who were 60 years and over (mean age = 72.8). Logistic regression analyses were performed. The results showed that participants with overall mistreatment (OR, 2.11; 95% CI, 1.83-2.43), psychological mistreatment (OR, 2.12; 95% CI, 1.78-2.51), physical mistreatment (OR, 1.82; 95% CI, 1.10-2.99), and financial exploitation (OR, 1.33; 95% CI, 1.11 – 1.60) were more likely to report more depressive symptoms. There was no significant association between sexual mistreatment and depressive symptoms (p = 0.07). Longitudinal studies are needed to obtain a more comprehensive understanding of the pathways between elder mistreatment and depressive symptoms.

